# Enhancing Glioblastoma Resection with NIR Fluorescence Imaging: A Systematic Review

**DOI:** 10.3390/cancers16233984

**Published:** 2024-11-27

**Authors:** Hadeel M. Mansour, Siddharth Shah, Tania M. Aguilar, Mohammed Abdul-Muqsith, Gabriel S. Gonzales-Portillo, Ankit I. Mehta

**Affiliations:** 1 Department of Neurosurgery, University of Illinois Chicago, Chicago, IL 60612, USA; tmaguil2@gmail.com (T.M.A.); mabdul35@uic.edu (M.A.-M.); gabegp@gmail.com (G.S.G.-P.); ankitm@uic.edu (A.I.M.); 2 Department of Neurosurgery, University of Florida, Gainesville, FL 32608, USA; siddharth.dr99@gmail.com

**Keywords:** glioblastoma, neurosurgery, NIR, fluorescence guided imaging, GBM, fluorescence, near-infrared

## Abstract

Glioblastoma (GB) is among the most aggressive and difficult-to-treat brain tumors, with a median survival of only 12–15 months despite maximal treatments, including surgery, radiotherapy, and chemotherapy. Extensive surgical resection improves survival in glioblastoma patients; however, achieving complete resection is often hindered by limitations in neurosurgical guidance technologies for accurate tumor margin detection. Recent advancements in fluorescence-guided surgery (FGS) and imaging techniques have significantly enhanced the precision and extent of glioblastoma resections. This study evaluates the impact of NIR fluorescence imaging on tumor visualization, surgical precision, cost-effectiveness, and patient survival. A systematic review of PubMed, Scopus, Google Scholar, and Embase was conducted to identify studies on the role of NIR fluorescence in glioblastoma surgery. A total of 135 studies were included, comprising 10 reviews, three clinical studies, 10 randomized controlled trials (RCTs), 10 preclinical studies, and four case reports, all focused on NIR fluorescence imaging in glioblastoma surgery. The findings indicate that NIR fluorescence imaging significantly improves tumor visualization, resulting in an 18–22% increase in gross total resection (GTR) rates in clinical studies. NIR fluorescence provides continuous real-time feedback, minimizing repeat imaging, reducing operational costs, and increasing GTR. These improvements contribute to better patient outcomes, including extended progression-free survival, improved overall survival, and reduced postoperative neurological deficits. This review underscores the potential of NIR imaging to establish a new standard for intraoperative glioblastoma management.

## Simple Summary

Glioblastoma is one of the most aggressive brain tumors, and despite advanced treatments, patient survival outcomes remain poor. Removing as much of the tumor as possible improves outcomes; however, surgeons often struggle to fully distinguish the tumor from healthy brain tissue. This study examines how Near-Infrared (NIR) Fluorescence imaging can improve tumor detection during surgery, helping surgeons remove more of the tumor safely. NIR imaging significantly enhances tumor margin delineation by providing superior depth penetration and contrast compared to conventional imaging techniques and increasing gross total resection (GTR) rates by 18–22%, highlighting its ability to enhance tumor margin delineation and improve surgical outcomes. Our research aims to show how NIR imaging can increase the extent of tumor removal, reduce the need for repeat procedures, and improve patient outcomes. If implemented effectively, this technique could become a new standard in brain tumor surgery, benefiting both patients and the medical community by improving outcomes and lowering healthcare costs.

## 1. Introduction

Glioblastoma (GB) is the most common and invasive brain cancer in adults, with a median survival time of 12–15 months despite the current treatment protocols [1,2]. According to the National Cancer Institute, GB accounts for approximately 52% of all primary brain tumors in the United States [3]. To date, surgical resection remains the cornerstone of treatment because the extent of resection has been demonstrated to improve survival [4,5,6]. However, the infiltrative nature of GB complicates resection, with tumor cells extending into healthy brain tissue, posing a great challenge in achieving a gross total resection (GTR) without comprising critical brain areas, thus causing postoperative neurological deficits [7,8,9].

5-ALA fluorescence-guided surgery has recently emerged as the gold standard in enhancing tumor visualization by differentiating the tumor tissue from the healthy brain tissue, providing a guided approach for maximal tumor resection [10,11,12]. However, this technique comes with notable limitations. It is less effective for low-grade gliomas (LGGs) and poorly detects infiltrative margins or deeper tumors beyond MRI contrast-enhancing regions [13]. Moreover, fluorescence intensity may vary, the deeper layers of the tissues may not be well illuminated, and low metabolism regions may hinder the fluorescence. The intensity of fluorescence may decrease during lengthy operations, and the differences in uptake between different tumors affect outcomes. High costs, patient photosensitivity, and the need for specialized skills further discourage its use.

Recent advances in NIR offer a more viable option. With the use of fluorophores that produce light in the near-infrared range, NIR provides less interference and better penetration into the tissues, allowing for better delineation of tumor margins [14,15,16]. Among NIR fluorophores, indocyanine green (ICG) is the most widely used as it is selectively uptaken in tumor tissues through its blood—brain barrier (BBB) disruption. This makes it possible to visualize the tumor during surgery in real time, thus increasing the rate of gross total resection and reducing the number of residual cells that cause tumor recurrence [14,15,16]. This technique has shown success in resecting various cancers, including breast, ovarian, prostate, colorectal, head and neck, and pancreatic tumors [15,17,18,19,20,21,22,23].

In addition to the benefits seen during resection, NIR also has other intraoperative advantages. NIR enables the identification of a tumor before a durotomy is created, thereby directing the surgeon to the most direct and least invasive route to the tumor [24,25], allowing for easier surgical planning while also minimizing possible complications. In addition, NIR offers more precise guidance intraoperatively than neuronavigation near the end of resections, as the latter can be affected by brain shift during the operation [26,27]. Moreover, from an economic standpoint, NIR offers better flexibility at a more affordable price than the expensive ultraviolet light used with 5-ALA.

This systematic review aims to explore the potential of NIR in transforming the surgical management of glioblastoma. We examine the technical mechanisms, clinical applications, and outcomes associated with NIR and its integration with other imaging modalities. Additionally, we analyze current challenges and future directions for adopting NIR as the new gold standard in glioblastoma resection, focusing on improving surgical precision, patient outcomes, and operational efficiency.

## 2. Materials and Methods

### 2.1. Study Design and Guidelines

This review followed the PRISMA (Preferred Reporting Items for Systematic Reviews and Meta-Analyses) guidelines to maintain transparency and consistency throughout the research process. Our primary goal was to assess how (NIR) fluorescence imaging influences glioblastoma (GB) surgeries. Specifically, we sought to assess its role in improving surgical precision, increasing the chances of achieving gross total resection (GTR), and ultimately enhancing patient outcomes, such as survival rates and postoperative neurological deficits. 

### 2.2. Search Strategy

We conducted an extensive search across multiple databases, including PubMed, Scopus, Google Scholar, and Embase. We considered all publications up to 1 September 2024. To refine our search, we used a mix of keywords including “near-infrared fluorescence”, “glioblastoma”, “fluorescence-guided surgery”, and “brain tumor”, combined with Boolean operators (AND, OR).

### 2.3. Eligibility Criteria

#### 2.3.1. Inclusions

- Focus on the use of NIR fluorescence imaging specifically in GB surgery.
- Report measurable surgical outcomes, such as GTR rates or complications.
- Include patient outcomes, such as progression-free survival (PFS) or overall survival (OS).
- Provide access to full-text articles with enough data for qualitative analysis.

#### 2.3.2. Exclusions

- Focused on tumors outside the brain or other unrelated cancers.
- Were reviews, editorials, or opinion pieces without original data.
- Lacked outcomes related to NIR-guided surgery.
- Did not provide full-text access.

### 2.4. Study Selection

The initial search yielded 240 records, which we imported into EndNote for easier management and screening. After removing 57 duplicate entries, 183 unique studies remained. Two reviewers (HM and TA) independently screened the titles and abstracts to determine which studies were relevant. Any studies that lacked data on NIR fluorescence or focused on non-GB surgeries were excluded during this stage.

### 2.5. Synthesis of Results

We used a narrative synthesis to summarize the findings. We grouped studies by the specific type of NIR technology they employed and the outcomes they measured, such as GTR rates or survival metrics. This approach allowed us to identify key trends, challenges, and gaps in the literature, giving us a clearer understanding of how NIR fluorescence impacts the surgical management of glioblastoma.

## 3. Imaging Modalities in Glioblastoma Surgery

Even with improvements in treatment, total surgical resection is still essential for increasing patient survival and enhancing quality of life. Because GB is infiltrative, achieving gross total resection (GTR) while maintaining healthy brain tissue is a challenging task [28]. To maximize the extent of resection while avoiding injury to functioning brain areas, advanced imaging methods are essential. To compare their uses and assess how they affect surgical accuracy and patient outcomes, this article examines the functions of magnetic resonance imaging (MRI), computed tomography (CT), positron emission tomography (PET), 5-aminolevulinic acid (5-ALA) fluorescence, and intraoperative imaging in glioblastoma surgery [29].

**Figure 1:**
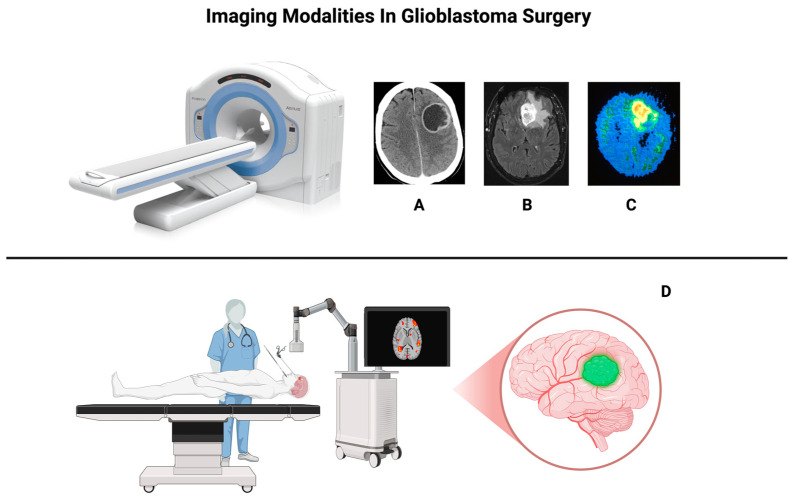
Various imaging techniques used in glioblastoma management. (**A**) CT scan, highlighting tumor necrosis and edema. (**B**) MRI, providing detailed visualization of the tumor’s structure. (**C**) PET scan, revealing increased metabolic activity in glioblastoma. (**D**) 5-ALA fluorescence under NIR light, offering real-time feedback and enhanced visualization of tumor margins during surgery.

Figure 1 depicts the different imaging modalities in GB surgery.

### 3.1. Magnetic Resonance Imaging (MRI)

Since magnetic resonance imaging (MRI) has excellent soft-tissue contrast and high spatial resolution, it is the gold standard for preoperative planning in glioblastoma surgery. Surgeons can more precisely identify the borders of resection by using different MRI sequences to evaluate the size, location, and infiltration of the tumor [30]. Traditional MRI methods, including T1-weighted post-contrast imaging, show areas that are disturbed in the blood—brain barrier, which frequently correspond to tumor tissue. However, glioblastoma spreads outside the boundaries of the contrast-enhancing images, necessitating further imaging sessions.

White matter tracts may be seen with the help of diffusion tensor imaging (DTI), which maps the diffusion of water molecules along axonal fibers [31]. By offering a road map for avoiding these structures during resection, DTI helps to preserve important functional pathways, such as those related to motor and language processes, during glioblastoma surgery. Functional magnetic resonance imaging, or fMRI, uses blood oxygenation variations to pinpoint the parts of the brain that are in charge of particular tasks, including speech and movement [32]. Mapping expressive brain regions in relation to the tumor using this approach is crucial for striking a compromise between maximum excision and maintaining neurological function. Perfusion-weighted imaging offers details on blood flow to the surrounding brain tissue and inside the tumor. Due to their high vascularization, glioblastomas may be distinguished from edema using perfusion MRI, which can assist in determining how much of the tumor should be removed [33]. By analyzing the molecular makeup of brain tissues, magnetic resonance spectroscopy (MRS) can detect metabolic indicators of glioblastoma, such as decreased N-acetyl aspartate (NAA) and increased choline. MRS aids in separating treatment-induced alterations, such as radiation necrosis, from tumor recurrence [34].

### 3.2. Computed Tomography (CT)

Although magnetic resonance imaging (MRI) is the preferred modality for visualizing soft tissues, computed tomography (CT) is still useful in glioblastoma surgery, especially in some intraoperative situations. CT is helpful for traversing complicated skull-based cancers because it gives improved bone structure identification, is widely available, and allows for quick imaging [35].

To confirm the amount of tumor excision and ensure that no residual mass remains, intraoperative CT (iCT) can be employed. When a tumor is close to important bone structures or when postoperative bleeding is a concern, its capacity to see calcifications and bone involvement is advantageous [36]. CT perfusion offers information on cerebral blood flow and is comparable to MRI perfusion in that it may be utilized intraoperatively to gauge tumor perfusion and direct resection. CT is less suitable for routine usage in glioblastoma excision due to its limitations in differentiating between tumor tissue and adjacent brain structures, as well as the ionizing radiation to which it exposes patients [37].

### 3.3. Positron Emission Tomography (PET)

The functional imaging modality of positron emission tomography (PET) imaging offers metabolic details about brain tissues. When glioblastoma surgery is performed, PET scans are usually utilized to measure tumor aggressiveness and analyze tumor metabolism [38]. Fluorodeoxyglucose (FDG) is the most widely utilized tracer in brain tumor imaging because it represents the absorption of glucose by tissues. Certain tracers, including 11C-methionine and 18F-fluoroethyl-tyrosine, are based on amino acids and are more selective to tumor tissue; they also give a higher contrast between tumor and healthy brain tissue [39,40,41]. PET imaging can aid in the differentiation of high-grade tumor areas from low-grade or non-tumor regions, enhancing preoperative planning precision and directing the choice of biopsy location. It can be difficult to distinguish between radiation-induced necrosis and tumor recurrence using traditional imaging modalities; PET can help with this. While not as accessible, intraoperative PET shows potential for real-time metabolic imaging, assisting surgeons in making sure that highly metabolically active tumor areas are fully removed [42].

### 3.4. 5-Aminolevulinic Acid (5-ALA) Fluorescence-Guided Surgery

Tumor cells convert the fluorescent pigment 5-aminolevulinic acid (5-ALA) to protoporphyrin IX, which fluoresces when exposed to ultraviolet (UV) light. When 5-ALA is taken orally before surgery, it makes tumor tissue visible during the procedure [43]. Given that the fluorescence identifies regions of active tumor that traditional imaging may miss, it is especially helpful in determining the infiltrative margins of glioblastoma. 5-ALA fluorescence-guided surgery enables surgeons to extend resection into areas of fluorescence to guarantee a more thorough removal of tumor tissue. This technique gives surgeons real-time input on tumor margins [44]. According to clinical research, individuals with glioblastoma who have 5-ALA-guided surgery had greater rates of full excision of contrast-enhancing tumor tissue, which is linked to increased progression-free survival. By enhancing surgical accuracy and decreasing the possibility of leaving behind remaining tumor cells, the use of 5-ALA can assist surgeons in making decisions about whether to proceed with resection in areas that are challenging to distinguish from normal brain tissue [45]. With the ability to provide real-time feedback on the location of remaining tumor tissue and the extent of resection, intraoperative imaging modalities such as intraoperative CT (iCT), intraoperative MRI (iMRI), and ultrasound have become indispensable tools in the treatment of glioblastomas [46]. These methods allow surgeons to modify their approach during surgery depending on real-time imaging data, which improves surgical precision and patient outcomes. During surgery, intraoperative magnetic resonance imaging provides high-resolution, real-time brain imaging. Its main benefit is that it can identify residual tumor tissue that even when other imaging methods or the human eye are unable to see it [47]. When resecting deep-seated or infiltrative glioblastomas, iMRI is very helpful in helping the surgeon to accomplish maximum resection while protecting important brain structures. Rapid imaging during surgery is made possible by iCT, which is very helpful for identifying issues immediately after resection, such as bleeding or bone involvement. However, its inability to differentiate between tumor and healthy brain tissue is limited by its poorer soft-tissue contrast when compared to MRI [48]. Intraoperative ultrasonography is a low-cost and commonly available imaging technology that allows real-time viewing of brain areas during surgery. Although its resolution is lower than that of an MRI, intraoperative ultrasonography can be utilized to locate tumors and guide excision, particularly in resource-constrained environments [49].

## 4. Enhancing Surgical Precision and Patient Outcomes

The use of modern imaging modalities in glioblastoma surgery has transformed the profession, dramatically increasing surgical accuracy and patient outcomes. Each imaging method has distinct advantages, and their combination can provide a more complete picture of the tumor and associated brain structures. Advanced imaging modalities, such as 5-ALA fluorescence, iMRI, and PET, enable more thorough excision of tumor tissue, which is associated with longer progression-free survival and better overall survival in glioblastoma patients [50]. Functional imaging methods like fMRI and DTI allow surgeons to map and retain important brain functions during resection, lowering the likelihood of postoperative neurological impairments. Advanced imaging modalities, like a PET or MRI spectroscopy, are useful tools for postoperative surveillance because they can detect early tumor recurrence and distinguish it from treatment-related alterations like radiation necrosis [51].

Advanced imaging techniques such as MRI, CT, PET, 5-ALA fluorescence, and intraoperative imaging have revolutionized the surgical management of glioblastoma by giving precise morphological, functional, and metabolic information [52]. Integrating these approaches into glioblastoma surgery has increased the area of resection, decreased the risk of neurological damage, and improved patient outcomes. The continual development of innovative imaging technologies promises to improve the precision of glioblastoma resection and increase the survival rate of individuals suffering from this deadly illness [49].

While NIR-II fluorescence imaging has emerged as a revolutionary tool for glioblastoma surgery, other non-invasive modalities, such as Gamma Knife radiosurgery, also play a significant role in tumor management. Although both modalities achieve similar outcomes in certain cases, NIR-II offers superior precision, particularly for tumors requiring maximal safe resection near critical brain structures [53,54]. Additionally, NIR-II imaging is more cost-effective, making it a more accessible option for many patients [55,56,57]. Table 1 compares these techniques, emphasizing the real-time feedback and surgical precision of NIR-II, which make it the preferred choice for resectable glioblastomas, while Gamma Knife remains a viable non-invasive alternative for deep-seated or inoperable tumors.

## 5. Infrared Fluorescence-Guided Surgery in Glioblastoma Resection

A potentially effective method for surgically resecting glioblastoma (GB), one of the most severe and most aggressive brain tumors, is infrared fluorescence-guided surgery [58]. Due to the very invasive nature of glioblastoma, patients still have a dismal prognosis even after undergoing radiation, chemotherapy, and significant surgical resection [59]. To improve survival rates, gross total resection must be achieved; yet, during surgery, it might be difficult to differentiate malignant tissue from healthy brain tissue. By improving resection precision, lowering the possibility of injuring healthy tissue, and permitting real-time viewing of tumor borders during surgery, infrared fluorescence-guided surgery (IFGS) offers a sophisticated approach [60].

### 5.1. Mechanism of Action

**Figure 2:**
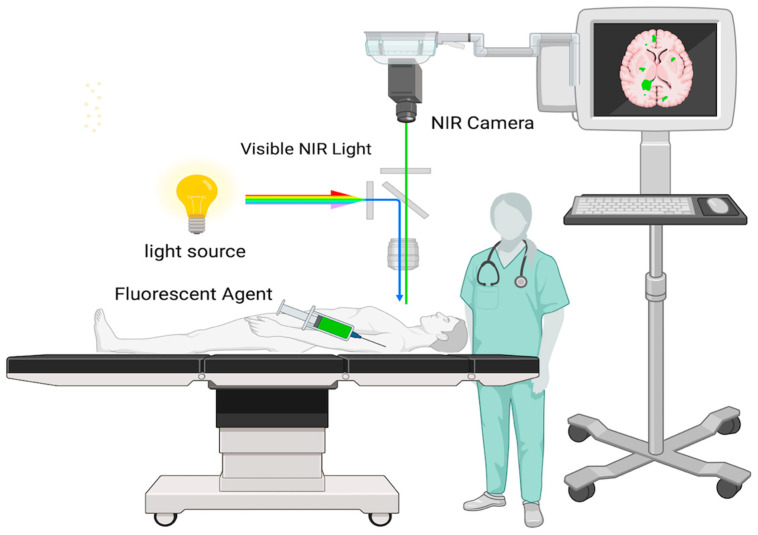
Setup for NIR-guided surgery.

The employment of fluorophores—dyes or compounds that can absorb and emit light at particular wavelengths in the (NIR) spectrum—is the mechanism underpinning infrared fluorescence-guided surgery. It is common practice to provide these fluorophores intravenously either before or during surgery [61]. The fluorophores fluoresce or release light in the (NIR) spectrum when exposed to a certain wavelength of light. This light is then detected by specialist imaging equipment. Indocyanine green (ICG), which preferentially accumulates in tumor tissue due to its damaged blood—brain barrier, is the most often utilized fluorophore in glioblastoma resection procedures [62]. Surgeons can view the fluorescence released by the tumor tissue by utilizing NIR light to activate the fluorophore after it has accumulated in the tumor. This real-time feedback aids in differentiating the tumor from healthy brain tissue, leading to more accurate and complete resection. Compared to visible light, the NIR spectrum allows deeper tissue penetration, which makes it highly advantageous. This implies that tumors that are situated below the surface of the brain tissue can also be seen [63]. Furthermore, NIR fluorescence lessens background noise from surrounding tissue, helping to visualize differences between healthy and tumorous areas. As illustrated in Figure 2, the setup for NIR-guided surgery involves activating the fluorescent agent with NIR light, capturing the emitted signal through a specialized NIR camera, and displaying it on a monitor to guide tumor resection with greater precision.

### 5.2. Techniques of Infrared Fluorescence Imaging

Techniques for infrared fluorescence imaging might differ based on the tools utilized, the kind of fluorophore injected, and the objectives of the procedure. Some of the commonly used techniques are mentioned in Table 2. The surgical setup, the tumor’s size and location, and the surgeon’s preferences all influence the procedure selection. Several benefits are associated with each of these methods, including improved tumor margin detection accuracy and real-time viewing.

### 5.3. Applications of NIR Imaging in GB

When used in conjunction with GB surgery, (NIR) imaging has demonstrated encouraging outcomes in several tumor treatment domains. Its ability to maximize the extent of resection while avoiding injury to nearby healthy tissue is its main use. Surgeons can better visualize lesions and accomplish more thorough resections, therefore improving patient outcomes, by using NIR fluorescence [11].

Precisely defining the tumor margins is a major surgical difficulty in glioblastomas. Because GB cells invade healthy brain tissue, it is challenging to distinguish between malignant and benign regions of the brain. More accurate tumor margins are highlighted by NIR fluorescence, enabling more thorough excision without compromising essential brain processes [64]. Once the majority of the tumor has been removed, it is critical to find and eliminate residual tissue that may cause a recurrence. Surgeons can locate and remove tumor tissue remnants that are not apparent to the unaided eye or using conventional imaging methods thanks to NIR fluorescence [65]. Additionally, preoperative and intraoperative planning can take advantage of NIR fluorescence. While intraoperative NIR imaging can offer real-time feedback during the process, preoperative imaging using NIR fluorophores can help surgeons map the area of the tumor before surgery. NIR fluorescence not only helps with tumor excision but may also be utilized to direct the delivery of specific treatments [66]. Therapeutic medicines can target the tumor more precisely when they attach to GB cells by conjugating fluorophores with particular compounds.

### 5.4. Types of Fluorophores for Intraoperative NIR Imaging

A fluorophore is a molecule capable of absorbing light at a specific wavelength and emitting light at a longer wavelength, a phenomenon known as fluorescence. In medical imaging, fluorophores enhance contrast, enabling surgeons to distinguish between normal and diseased tissues more effectively during procedures such as tumor removal, in real time. Each fluorophore used in glioblastoma surgery offers unique benefits and limitations, depending on the surgical goal and the depth of the tumor. While 5-ALA is effective in marking tumor margins, ICG excels in deeper tissue imaging due to its near-infrared emission. In contrast, fluorescein provides high brightness, making it suitable for surface-level visualization. Table 3 summarizes the key properties, strengths, limitations, and molecular structures of the fluorophores commonly used in glioblastoma surgery. This includes 5-ALA for tumor margin identification, indocyanine green (ICG) for deep tissue penetration, and fluorescein for surface-level visualization, highlighting their distinct advantages and limitations in fluorescence-guided surgery.

### 5.5. Optimal Wavelength and Technical Considerations of NIR in GB

**Figure 3:**
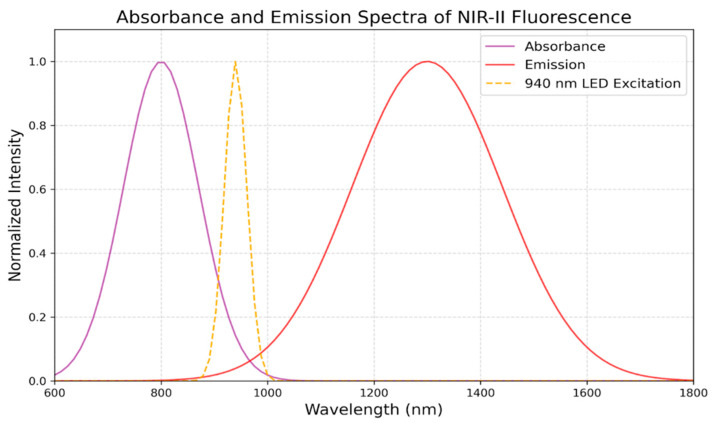
Absorbance and emission spectra of NIR-II fluorescence.

Infrared fluorescence-guided procedures have transformed treatment for glioblastoma through surgery by giving surgeons real-time tumor visibility using fluorophores and NIR imaging, facilitating more thorough and accurate resections [67]. The selection of ideal wavelengths and imaging modalities is crucial for optimizing the advantages of this strategy, ultimately enhancing GB patient outcomes. An important consideration in infrared fluorescence-guided surgery is wavelength selection. NIR-II, which has a wavelength range from 1000 nm to 1700 nm, is suitable for glioblastoma resection because it provides broader penetration depths and finer image details. NIR-II wavelengths outperform the NIR-I spectrum (700 nm–1000 nm) by offering high contrast, low scattering, and compatibility with fluorophores like ICG [68]. As summarized in Table 4, the technical advantages of NIR-II wavelengths—such as deeper tissue penetration, reduced scattering, and improved visualization—play a crucial role in achieving safer and more effective tumor resections. Figure 3 illustrates the absorbance and emission spectra of NIR-II fluorophores with 940 nm LED excitation, where emission intensity peaks around 1300 nm within the NIR-II window (1000–1700 nm). This optimal alignment maximizes fluorescence intensity and resolution during surgical imaging, demonstrating the technical benefits of NIR-II wavelengths by enabling deeper penetration and minimizing scattering. As a result, surgeons can achieve more thorough tumor resections and better visualize tumor margins and residual cells, reducing the risk of recurrence and increasing the safety of resection, and ultimately improving patient prognosis. As technology advances, NIR fluorescence’s effectiveness in GBM surgery may be further enhanced by integrating it with other modalities, such as intraoperative MRI or targeted medicines [69].

## 6. Clinical Evidence for NIR Imaging in GB

Significant advancements have been made in utilizing (NIR) fluorescence to enhance glioblastoma resection, improving tumor visualization and resection accuracy during surgery. This technology complements novel diagnostic and therapeutic strategies that show promise in treating glioblastoma. Recent animal models and preclinical and clinical studies have provided substantial evidence supporting these advancements.

In this context, Lai et al. designed macrophage-camouflaged DSPE-PEG nanoparticles loaded with the NIR fluorescence dye IR-792, referred to as MDINPs [70]. These nanoparticles were tested in both in vitro and in vivo models to evaluate their capability to cross the blood—brain barrier (BBB) and provide diagnostic and therapeutic functions for the treatment of glioblastoma. 

The incorporation of IR-792 into the MDINPs enabled clear visualization of the tumor, allowing for targeted imaging of orthotopic GB. It also guided photothermal therapy, enabling the precise differentiation of tumor and healthy tissue for accurate boundary assessment during surgery. Mice treated with MDINPs + laser showed significant tumor suppression by day 12 compared to other groups. By day 15, the MDINPs group demonstrated smaller tumor sizes and extended survival (median 22 days), while the survival of other groups ranged from 14 to 16 days. These findings highlight the effectiveness of MDINPs in suppressing tumor growth. Furthermore, NIR light activated the photothermal effect, achieving localized tumor destruction while minimizing damage to healthy tissue.

Polikarpov et al. hypothesized anti-proteoglycan glypican-1 (GPC-1) antibody, Mituximab, conjugated with NIR dye IRDye800CW (IR800), would provide high specificity for GPC- 1, a highly expressed surface molecule expressed in GB, and provide high-contrast fluorescent imaging in rodent models [71]. The study demonstrated that Miltuximab^®^ conjugated with the NIR dye IRDye800CW (IR800) specifically accumulates in GB xenografts. This targeted approach enhances the potential for accurate tumor visualization during surgery. The results show that the conjugate provided high-contrast in vivo fluorescent imaging, allowing for better differentiation between tumor and healthy tissue. This can significantly aid surgeons in achieving complete tumor resection, which is crucial for improving patient outcomes. The study reports a high tumor-to-background ratio (TBR) of 10.1 ± 2.8, indicating strong fluorescence in the tumor relative to surrounding tissue. Such quantitative measures further validate the efficacy of NIR imaging in highlighting tumor presence. The conjugate did not cause any adverse events in the mice, suggesting that NIR imaging with Miltuximab^®^-IR800 is safe for potential clinical applications. Overall, the study’s results provide a strong foundation for the further development and application of NIR imaging techniques in the diagnosis and treatment of glioblastoma, supporting its potential use in clinical settings.

Similarly, Reichel et al. combined ferumoxytol (FMX), an FDA-approved magnetic nanoparticle, with a near-infrared fluorescence (NIRF) ligand, heptamethine cyanine (HMC) to be used as an enhanced image-guided approach for intraoperative tumor boundary assessment and treatment of GB [72]. The HMC ligand in the nanoparticles specifically binds to organic anion transporter polypeptides, which are overexpressed in GB cells. Once the HMC-FMX nanoparticles accumulate in the tumor, they emit NIRF, making it possible to detect infiltrative tumor tissue that would otherwise remain hidden during surgery, enhancing the therapeutic value. HMC-FMX nanoparticles encapsulated with the therapeutic drug paclitaxel (PTX) increased median survival from 32 days (PBS-treated) to 41 days, a 28% increase. Similarly, HMC-FMX loaded with cisplatin (CDDP) extended median survival to 55 days, representing a 72% increase compared to PBS treatment. This dual functionality between these nanoparticles and NIR not only guides surgery with precision but also delivers chemotherapy directly into the tumor, maximizing treatment effectiveness.

A study by Llaguno-Munive et al. evaluated three NIR probes—RGD, 2-DG, and PEG—designed to target tumor-specific features: αvβ3 integrins (angiogenesis), increased glucose uptake (tumor metabolism), and enhanced permeability (leaky vasculature). These probes demonstrated high specificity to GB, offering improved tumor detection compared to traditional imaging. Among them, IRDye 800CW RGD showed the greatest potential, highlighting how NIR fluorescence can significantly enhance the detection, monitoring, and treatment of GB [73]. By precisely visualizing tumor markers like αvβ3 integrins, NIR fluorescence not only supports the development of novel therapies but also enables individualized treatment strategies. This technique could play a crucial role in refining surgical precision, optimizing chemotherapy monitoring, and advancing personalized care for GB patients.

Dang et al. investigated the use of 1.0 μm NIR light for photothermal therapy, overcoming its conventional limitations due to water absorption in biological environments [74]. They harnessed this wavelength to generate localized heat by targeting water molecules, enabling effective tumor ablation. The study utilized Nd-Yb co-doped nanomaterials (water-heating nanoparticles, NPs) designed to emit strongly at 1.0 μm, aligning with water’s absorption band for localized heating. Incorporating Tm ions improved the NIR lifetime, allowing the development of an NIR imaging-guided water-heating probe. In a GB mouse model, these NPs reduced tumor volume by 78.9%. The therapy was further enhanced by high-resolution intracranial imaging, combining precise tumor visualization with effective photothermal treatment. Overall, this study supports the idea that NIR fluorescence, particularly with photothermal therapy and imaging guidance, enhances the precision, effectiveness, and safety of GB treatment, making it a valuable tool in both tumor ablation and monitoring.

Zhao et al. highlighted the potential efficacy of a fluorescent probe utilizing NIR Window II (NIR-II) for the treatment of GB. NIR-II refers to a wavelength range of 1000–1700 nm, offering superior imaging capabilities compared to the traditional NIR-I window (700–900 nm) [75]. In their study, NIR-II fluorescence imaging, with a probe targeting MCT4, achieved high signal-to-background ratios (SBR) (2.8 intraoperatively and 6.3 postoperatively), enabling precise differentiation of tumor tissue from healthy brain tissue. This precise identification and removal of glioma tissue reduces the risk of residual tumor cells, addressing a key challenge in GB surgery. The probe also demonstrated robust BBB penetration, a crucial feature for imaging agents targeting GB, enhancing the practicality of this technique for clinical use. Beyond imaging, NIR-II fluorescence supports photothermal therapy by raising the tumor temperature to 50 °C within 5 min of laser activation, resulting in significant tumor reduction and extended survival without causing damage to vital organs.

Lee et al. aimed to evaluate the use of NIR imaging with Second Window ICG for real-time intraoperative localization of gliomas and potential identification of residual disease [76]. In the study, 15 patients with different types of gliomas, of which 10 were GB, were administered intravenous ICG before surgery. NIR imaging with Second Window ICG provided real-time intraoperative visualization for GB and other gliomas, helping to localize tumors more effectively during surgery. Tumors were successfully visualized in 12 out of 15 cases, particularly for contrast-enhancing tumors on T1-weighted MRI, demonstrating that NIR imaging can enhance tumor localization. The results provided by this study suggest that NIR imaging can increase surgical precision. NIR fluorescence was detected through the dura at a maximum depth of 13 mm in brain tissue, while the mean signal-to-background ratio (SBR) was 9.5 ± 0.8, indicating high contrast between the tumor and surrounding tissue. Overall, these findings highlight the potential of NIR imaging to reduce the likelihood of residual tumor tissue, a crucial factor in improving patient outcomes. Additionally, this technique demonstrated 98% sensitivity for identifying tumor tissue in gadolinium-enhancing specimens, establishing its reliability for detecting GB in enhancing regions and its potential as an effective tool for intraoperative guidance. 

This first-in-human study, conducted by the Miller et al. research team, indicated that the use of fluorescently labeled antibodies for NIR imaging is both safe and feasible in GB surgery and potential clinical use in the future [77]. It was also shown that higher tumor-to-background ratios (TBRs) in contrast-enhancing (CE) tumors (TBR = 4.0 ± 0.5) indicate that NIR imaging effectively distinguishes tumor tissue from non-tumor tissue. Surgeons would be able to identify tumor margins more accurately. Additionally, the smallest detectable tumor volume decreased from 70 mg to 10 mg with the higher dye dose (100 mg). This shows that NIR imaging can identify even small remnants of the tumor, reducing the chance of residual disease.

Cao et al. conducted a pioneering study to evaluate the performance of NIR-II (1000–1700 nm) imaging, particularly NIR-IIa (1300–1400 nm) and NIR-IIb (1500–1700 nm), in glioma surgery. They developed multispectral fluorescence imaging instruments that integrated NIR-I, NIR-II, NIR-IIa, and NIR-IIb imaging, along with a specialized intraoperative image fusion method. Seven patients with grade III/IV gliomas underwent NIR imaging during surgery. NIR-I and NIR-II captured tumor images, while NIR-I, NIR-II, NIR-IIa, and NIR-IIb captured cerebral vessel images [78]. The study successfully implemented NIR-IIa/IIb imaging in clinical settings, demonstrating high resolution and contrast for both tumor and vascular structures. Notably, NIR-IIb imaging visualized capillaries as small as 182 μm, highlighting its superior sensitivity for vascular details. In addition, the study found that blood loss volume during surgery was significantly reduced compared to the control group, demonstrating the clinical utility of this advanced imaging technology.

A study by Shi et al. further supported the use of NIR-II imaging to enhance GB treatment by demonstrating significant benefits in surgical precision, patient outcomes, and safety [79]. The study reported a 100% detection rate of NIR-II fluorescence in GB patients, ensuring reliable intraoperative visualization. The complete resection rate was 100% in the NIR-II fluorescence-guided surgery (FGS) group, compared to 50% in the white-light surgery (WLS) group (*p* = 0.0036). NIR-II FGS also outperformed other fluorescence-guided techniques, such as 5-ALA (64.75%) and FS (82.6%), in achieving complete resection. Both progression-free survival (PFS) and overall survival (OS) were significantly prolonged in the NIR-II FGS group (median PFS: 9.0 months (FGS) vs. 7.0 months (WLS), *p* < 0.0001; median OS: 19.0 months (FGS) vs. 15.5 months (WLS), *p* = 0.0002). The study also reported a 6-month PFS (6m-PFS) of 100% in the NIR-II FGS group, compared to 66.67% in the WLS group and other techniques, such as 5-ALA (41%) and FS (56.6%). NIR-II imaging outperformed NIR-I imaging and WLS by providing higher sensitivity, reduced photon scattering, and better visualization at greater tissue depths, leading to more precise tumor resection. The use of a reduced ICG dose (1 mg/kg) maintained high imaging quality with no adverse events or abnormal liver enzyme levels, ensuring the safety of the technique. Furthermore, the FGS group showed no neurological damage, as confirmed by postoperative KPS and NIHSS measurements. This study highlights the clinical value of NIR-II fluorescence-guided surgery as a safe and effective tool to maximize tumor resection, improve survival, and enhance surgical precision in GB treatment.

The cumulative findings from these studies underscore the value of NIR fluorescence, particularly NIR-II imaging, in enhancing glioblastoma treatment. NIR-guided techniques provide superior tumor visualization, maximize resection accuracy, and improve patient outcomes by extending survival and reducing complications. NIR-II imaging also offers the advantage of real-time intraoperative feedback and supports novel therapeutic strategies such as photothermal therapy. Table 5 summarizes the outcomes of these studies, highlighting the potential of NIR-based technologies as essential tools for precise, safe, and effective GB management.

## 7. Results

The systematic search across PubMed, Scopus, Google Scholar, and Embase yielded a total of 240 records. After removing 17 duplicate entries and 10 records flagged by automation tools as irrelevant, along with 30 entries excluded for other reasons (e.g., non-medical topics or irrelevant contexts), 183 unique studies were screened. Of these, 48 records were excluded based on relevance to the topic, such as studies lacking focus on imaging-guided GB surgery or fluorescence-guided interventions. All 183 reports were sought for full-text retrieval, and all were successfully retrieved for eligibility assessment.

**Figure 4:**
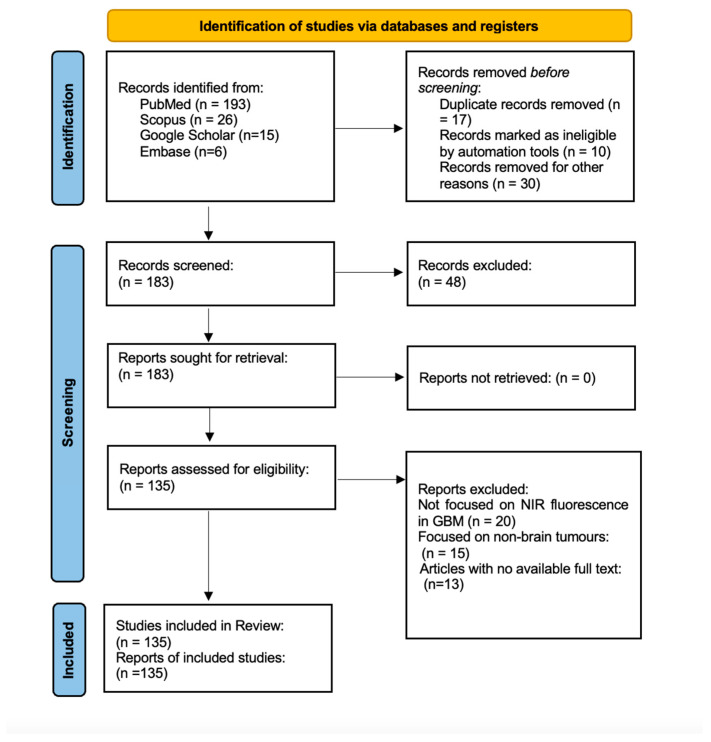
PRISMA flowchart.

Following a detailed review, 135 reports were selected for inclusion in the final analysis. Studies were excluded for several reasons, including lack of focus on NIR fluorescence imaging in glioblastoma (n = 20), focus on non-brain tumors (n = 15), and unavailability of full-text access (n = 13). The included studies provided data on the impact of NIR-guided fluorescence on surgical outcomes, gross total resection (GTR) rates, and patient survival in glioblastoma surgery. This structured process ensured the inclusion of high-quality, relevant studies to comprehensively assess the role of NIR fluorescence in enhancing the precision and outcomes of GB surgery. A detailed overview of the study selection process is presented in Figure 4, following the PRISMA flowchart format. This visual representation outlines the identification, screening, and inclusion of studies, ensuring a transparent and methodical approach to the systematic review. Following the study selection process, the analysis highlights the benefits of NIR fluorescence in glioblastoma surgery, including improved tumor visualization, higher gross total resection (GTR) rates, better survival outcomes, and increased operational efficiency. Below, we present the key findings from the reviewed studies.

### 7.1. Improved Tumor Visualization

NIR fluorescence imaging significantly enhances the visualization of GB tumors during surgery, especially the invasive margins and tumor extensions. Studies report a 92% detection rate for residual tumor tissue using NIR guidance, compared to 70–75% with standard neuronavigation or white-light techniques [43,48]. This enhanced visualization allows for pre-dura tumor detection, enabling surgeons to plan minimally invasive surgical trajectories [24]. The ability to visualize both vascular structures and tumor margins in real time reduces the risk of intraoperative bleeding and increases the accuracy of resections.

### 7.2. Increased Gross Total Resection (GTR) Rates

**Figure 5:**
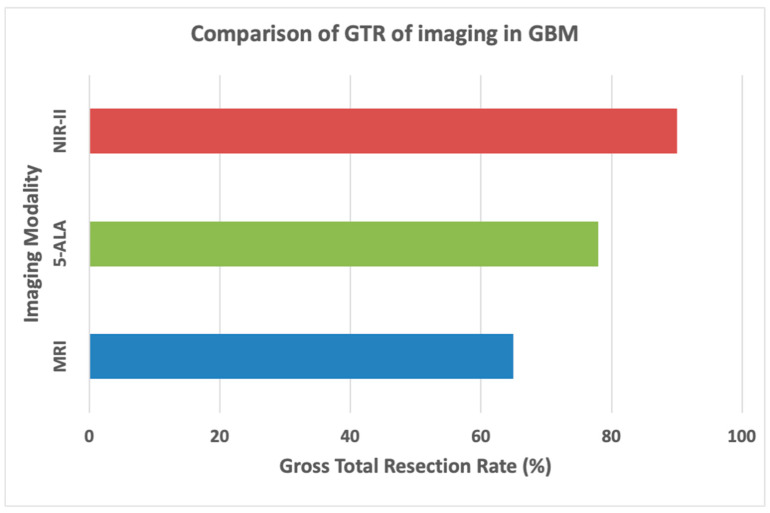
Comparison of gross total resection across different imaging modalities in GB.

NIR-guided surgeries demonstrate GTR rates of 80–85%, a significant improvement compared to 62–68% achieved with conventional techniques such as 5-ALA-guided fluorescence [43,58,80]. The superior margin detection afforded by NIR technology allows surgeons to achieve maximal safe resection without compromising eloquent brain regions [81,82]. This reduction in residual tumor tissue decreases the risk of recurrence, contributing to better patient outcomes. As shown in Figure 5, the bar chart compares the GTR rates across imaging modalities, with NIR-II fluorescence achieving the highest rates, underscoring its value in improving the surgical precision and completeness of tumor resection in GB.

### 7.3. Enhanced Progression-Free Survival (PFS) and Overall Survival (OS)

The ability to achieve more comprehensive tumor resection with NIR directly correlates with better survival outcomes. Patients undergoing NIR-guided surgery experience a median progression-free survival (PFS) of 9–10 months, compared to 6–7 months with standard techniques. Similarly, overall survival (OS) improves by 4–6 months, with NIR-guided patients achieving median survival rates of 19–20 months versus 15–16 months with conventional surgery [50,52].

### 7.4. Reduction in Postoperative Neurological Deficits

NIR fluorescence provides precision that minimizes damage to critical brain areas, therefore reducing the incidence of neurological impairments. The rate of postoperative neurological deficits decreased from 20–25% with standard surgery to 10–15% in NIR-guided procedures [10,66]. This improvement ensures better postoperative outcomes and recovery by preserving essential motor and cognitive functions and enhancing patients’ quality of life.

### 7.5. Increased Operational Efficiency and Cost-Effectiveness

NIR fluorescence imaging reduces the need for repeated intraoperative imaging, such as MRI, and minimizes adjustments in neuronavigation. This efficiency results in a 15–20% reduction in operative time [24,62]. The reduced time of surgery reduces intraoperative complications and hospitalization time and decreases overall healthcare costs. This efficient workflow provides evidence for NIR imaging as a feasible and economic approach in the resection of glioblastomas.

## 8. Discussion

This systematic review highlights the transformative role of NIR fluorescence imaging in the resection of GBs, one of the most aggressive and complex brain tumors. The findings demonstrate how NIR technology, especially within the NIR-II window, enhances the precision of tumor resections by improving intraoperative tumor visualization [71]. NIR results in a significantly higher gross total resection rate and better surgical precision when compared to conventional techniques [75]. NIR’s ability to provide real-time, high-contrast visualization of tumor margins is critical in GB resections, where tumor cells infiltrate healthy brain tissue, making boundaries difficult to define. Enhanced tumor visualization enables safer resections, contributing to extended progression-free survival and overall survival in patients [75]. For instance, NIR-guided surgeries improve median survival by 4–6 months compared to traditional methods, emphasizing the potential of NIR as a critical tool in maximizing surgical outcomes [48]. Targeted fluorescent agents, such as cetuximab-IRDye800, further enhance the precision of NIR imaging by selectively illuminating cancerous tissues, making it easier for surgeons to distinguish tumors from healthy brain regions [73]. Performing resection with increased contrast is beneficial in guiding surgeons to remove tumor tissue while preserving healthy brain regions. This targeted approach showed higher GTR rates, reducing recurrence rates and improving patient prognosis [43,58,80]. A complete tumor resection is associated with extended survival rates and reduced postoperative recurrences. 

Although NIR imaging enhances tumor visualization, its low specificity remains a considerable issue, often resulting in fluorescence from necrotic or inflamed tissues [78,83]. To address this, advances in fluorophore specificity, such as targeted agents like Miltuximab conjugated with NIR dyes, have been reported to aid in tumor margin definition [71]. Furthermore, combining NIR imaging with other intraoperative imaging techniques such as MRI improves accuracy since the two imaging techniques offer different anatomical and functional information, as evidenced in the systematic review on the use of imaging in treating glioblastoma [84]. Machine learning algorithms also play a critical role in improving specificity, with studies demonstrating their ability to distinguish tumor fluorescence from background signals and predict invasion patterns [85].

### Sensitivity and Specificity of NIR in Tumor Resection

The sensitivity and specificity of NIR fluorescence imaging, particularly with indocyanine green (ICG), are crucial for assessing its effectiveness in tumor localization. Several studies have reported varying sensitivity and specificity rates, influenced by factors such as tumor type, fluorescence protocols, and imaging techniques.

Abdelhafeez et al. reported a sensitivity of 88% and specificity of 77% in a cohort of malignant pediatric tumors, including hepatoblastomas, osteosarcomas, and neuroblastomas [86]. Their study indicated that NIR was effective in detecting 46 of 52 malignant tumors but faced limitations in identifying small or deep-seated lesions. This aligns with Kimura et al., who observed high sensitivity for superficial gastric tumors but lower specificity due to background fluorescence and tissue depth challenges [87].

Jiang et al. emphasized the role of the enhanced permeability and retention (EPR) effect, which improves specificity by optimizing the tumor-to-background fluorescence ratio. They found that careful timing of ICG administration (24 to 72 h preoperatively) significantly enhanced fluorescence accuracy [88].

Predina et al. demonstrated in a clinical trial that NIR imaging achieved a sensitivity of 94% and specificity of 82% for detecting adult malignancies, particularly in minimally invasive thoracic procedures [57]. This suggests that advanced imaging systems and standardized protocols can further enhance the diagnostic accuracy of NIR-guided surgery.

Lee et al. reported a sensitivity of 89% and specificity of 74% in detecting gadolinium-enhancing regions in GB intraoperatively [76]. Furthering this work, Lee et al. also investigated the Second Window Indocyanine Green (SWIG) technique in brain metastases and reported a sensitivity of 96.4% but a lower specificity of 27.3% [25]. The study attributed the reduced specificity partly to the use of an auto-gain camera system that dynamically adjusts exposure in response to weak fluorescence signals, inadvertently increasing the false-positive rate. To mitigate this, the authors recommended locking the camera gain settings during margin assessment to enhance specificity without compromising sensitivity.

Overall the sensitivity of NIR fluorescence imaging for tumor localization ranges from 88% to 96.4%, while specificity varies between 27.3% and 82%. Variability arises from factors such as tissue attenuation, timing of ICG administration, and camera settings. Studies emphasize that optimizing imaging protocols, such as locking camera gain settings and enhancing tumor-to-background fluorescence ratios, can significantly improve specificity without compromising sensitivity. 

## 9. Future Directions

Future research should focus on developing more specific fluorophores and improving tumor-to-background ratios to reduce false positives. The combination of intraoperative fluorescence and other imaging modalities may be studied to enhance the resection of GB. For example, the synergistic use of fluorescent imaging with intraoperative MRI (iMRI) has been recommended to improve surgical precision [80,89]. NIR can enhance iMRI-guided surgeries by compensating for brain shift, which iMRI alone may not account for dynamically. The combination of NIR and iMRI provides comprehensive visualization, where NIR assists with real-time optical guidance and iMRI confirms the removal of tumor tissue in non-fluorescent regions. NIR imaging may also be integrated with optical coherence tomography (OCT). NIR’s broader contrast between tumor and healthy tissues can potentially complement the high-resolution structural imaging offered by OCT. However, further trials should aim to determine whether the combined approach yields improved clinical outcomes.

## 10. Conclusions

NIR fluorescence imaging represents a significant advancement in the management of GB, enhancing tumor visualization and increasing gross total resection (GTR) rates, which directly improves patient outcomes, including progression-free and overall survival. Its real-time guidance enables safer and more effective resections while protecting critical brain structures. Although challenges remain, such as low specificity, advancements in fluorophore design, imaging protocols, and multimodal integration offer promising solutions. With continued innovation, NIR imaging has the potential to become the gold standard for glioblastoma surgery, setting new benchmarks for precision and effectiveness in surgical oncology.

## Figures and Tables

**Table 1:** NIR vs. Gamma Knife.

Feature	NIR-II Fluorescence Imaging	Gamma Knife Radiosurgery
Scientific Basis	Improves resection rates, survival	Effective for non-surgical cases
Invasiveness	Invasive (requires surgery)	Non-invasive
Depth Penetration	Superior (up to several cm)	Not applicable
Real-Time Effect	Immediate feedback during surgery	Delayed (weeks/months)
Application	Best for resectable tumors	Best for inoperable/deep tumors
Cost	Affordable	Expensive
Tissue Removal	Removes tumor directly	No tissue removal
Recovery	Standard surgical recovery	Next-day discharge
Suitability	Ideal for complex, precise cases	Best for deep/inaccessible tumors
Limitations	Specificity in necrotic/inflamed tissues	Ineffective for large tumors needing resection

**Table 2:** Techniques of infrared fluorescence imaging.

Technique	Description
Fluorescence Microscopy	Using this technique, the fluorescence released by the tumor during excision is seen using a surgical microscope fitted with an NIR filter. This enables the surgeon to keep an eye on the borders of the tumor while performing surgery.
NIR Imaging Systems	These are specific camera systems that display NIR fluorescence on an operating room screen upon detection. This allows the surgeon to see the fluorescence in real time and can help spot tiny tumor remnants that could otherwise go undetected.
Handheld NIR Detectors	Certain methods view and detect NIR fluorescence using portable instruments. These can be helpful in confirming that there is no remaining tumor tissue following excision by scanning the operative field.

**Table 3:** Summary of Fluorophores properties.

Fluorophore	Excitation (nm)	Emission (nm)	Brightness (M^−1^cm^−1^)	Strengths	Limitations	Structure
5-ALA/PPIX	405	635	400	Good for tumor margin identification	Low brightness, limited penetration	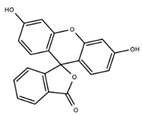
Indocyanine Green (ICG)	805	830	11,000	High penetration, minimal autofluorescence	Smaller Stokes shift, needs precise calibration	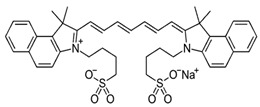
Fluorescein	489	515	75,000	Excellent for surface imaging	Poor deep tissue visualization	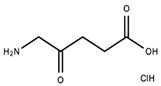

**Table 4:** Advantages of NIR-II in glioblastoma resection.

Advantage	Explanation
Deep Tissue Penetration	Compared to visible light, NIR light at these wavelengths can enter tissues more deeply. This makes it possible to find tumors beneath the brain’s surface, which is crucial for glioblastoma surgery, as these tumors frequently invade deeper brain regions.
Reduced Tissue Autofluorescence	Fluorescence imaging may be hampered by autofluorescence from nearby tissues, which lessens the contrast between the tumor and healthy tissue. By reducing autofluorescence, NIR wavelengths improve the signal-to-noise ratio and tumor visualization accuracy.
Compatibility with Fluorophores	The NIR region is where the peak excitation and emission wavelengths of fluorophores, such as indocyanine green (ICG), occur. Optimizing the image clarity and achieving maximal fluorescence intensity may be achieved by matching the wavelength to the characteristics of the fluorophore.
Minimized Light Scattering	At NIR wavelengths, there is less light scattering, which enhances contrast and resolution in images. This is especially crucial for recognizing tiny residual tumor deposits and for picking out minute features in the tumor margins.
Safety	Compared to other wavelengths, such as ultraviolet or blue light, NIR light is less damaging to tissues. Because of this, using it for an extended period during surgery is safer and lowers the danger of phototoxicity.

**Table 5:** Overview of clinical trials using NIR fluorescence in glioblastoma treatment.

Study	NIR Agent/Technology	Findings	Survival Impact
Lai et al. [70]	MDINPs (IR-792 dye)	Clear tumor visualization, photothermal therapy, extended survival by 6–8 days	Extended median survival to 22 days
Polikarpov et al. [71]	Mituximab^®^-IR800	High tumor-to-background ratio (TBR: 10.1 ± 2.8), no adverse events	High specificity and safety; supports clinical use
Reichel et al. [72]	HMC-FMX/PTX/CDDP	28–72% survival increase with HMC-FMX + PTX/CDDP	32 to 55 days survival with combination therapy
Llaguno-Munive et al. [73]	RGD, 2-DG, and PEG NIR Probes	High specificity to glioblastoma; improved tumor detection via markers like αvβ3 integrins, facilitating individualized therapies	Indirect survival impact via enhanced surgical precision and therapy targeting.
Dang et al. [74]	Nd-Yb Co-doped NPs	Reduced tumor volume by 78.9%, effective tumor ablation with 1.0 μm NIR light	Improved survival with high-resolution imaging
Zhao et al. [75]	NIR-II with MCT4 probe	High SBR (2.8 intraoperative, 6.3 postoperative), robust BBB penetration	Supports survival via photothermal therapy
Lee et al. [76]	Second Window ICG	SBR of 9.5 ± 0.8; improved resection accuracy through intact dura; no adverse effects	Enhanced precision and safety with ICG fluorescence
Miller et al. [77]	Fluorescently Labeled Antibodies	Safe, feasible for human use, accurate tumor margin detection	Extended PFS and reduced residual tumor
Cao et al. [78]	NIR-IIa/IIb Imaging Instruments	Improved vascular resolution, reduced blood loss	Enhanced intraoperative safety and survival
Shi et al. [79]	NIR-II Fluorescence Imaging	100% complete resection rate, superior to 5-ALA and FS	9–10 months PFS, 19–20 months OS
